# Factors Affecting Psoas Muscle Mass Index in Patients Undergoing Peritoneal Dialysis

**DOI:** 10.7759/cureus.56347

**Published:** 2024-03-17

**Authors:** Momoko Hirata, Kiyonori Ito, Susumu Ookawara, Keisuke Tanno, Junki Morino, Saori Minato, Yuko Mutsuyoshi, Taisuke Kitano, Keiji Hirai, Yoshiyuki Morishita

**Affiliations:** 1 First Department of Integrated Medicine, Saitama Medical Center, Jichi Medical University, Saitama, JPN

**Keywords:** vitamin d, serum albumin concentration, sarcopenia, psoas muscle mass index, peritoneal dialysis, computed tomography

## Abstract

Introduction: Many patients with chronic kidney disease (CKD), including peritoneal dialysis (PD), have sarcopenia. It is important to evaluate muscle mass to prevent sarcopenia in the field of CKD management. Recently, muscle mass assessment using psoas muscle evaluated by computed tomography (CT) has been reported in patients undergoing hemodialysis. However, few clinical studies have investigated the clinical factors associated with the evaluation of psoas muscle in patients undergoing PD.

Methods: Psoas muscle mass index (PMI) was measured in cross-sectional areas of the bilateral psoas muscles at the third lumbar spine level to evaluate psoas muscle status. The associations between PMI and possible clinical factors were investigated in 68 patients undergoing PD.

Results: The mean PMI was 6.3 ± 2.0 cm^2^/m^2^, and the PMI was higher in men than in women (p < 0.001). In a multivariable linear regression analysis of the factors associated with PMI, male gender (standardized coefficient: 0.331), body mass index (standardized coefficient: 0.283), serum creatinine concentration (standardized coefficient: 0.289), serum albumin concentration (standardized coefficient: 0.235), and the use of vitamin D (standardized coefficient: 0.195) were independently identified.

Conclusion: PMI was independently and significantly associated with gender, BMI, serum creatinine concentration, serum albumin concentration and the use of vitamin D. Further prospective studies are needed to clarify whether the maintenance of nutritional status or vitamin D administration could affect muscle mass in patients undergoing PD.

## Introduction

Sarcopenia is characterized by a decline of skeletal muscle, low muscle strength, and deteriorated physical performance [1]. As chronic kidney disease (CKD) progresses, the prevalence of sarcopenia increases [2]. Various factors, including increased chronic inflammation, metabolic acidosis, and malnutrition, could cause decrease in skeletal muscle mass in patients with CKD [3,4]. Furthermore, low skeletal muscle mass was reportedly associated with poor prognosis in patients undergoing dialysis [5]. Therefore, evaluation of muscle mass is essential in patients with CKD, including those on peritoneal dialysis (PD).

Recently, the measurement of the psoas muscle area using computed tomography (CT) scans has been commonly used as a simple validated clinical tool for muscle mass assessment [6,7]. In patients with end-stage renal disease (ESRD), CT scans are sometimes performed for intra-abdominal evaluation, including preoperative evaluation or annual evaluation for acquired cystic kidney disease and renal carcinoma. In patients undergoing hemodialysis (HD), psoas muscle mass index (PMI) evaluated by CT is positively correlated with skeletal muscle mass [8,9] and is a prognostic factor [7]. Additionally, PMI was affected by several factors, including sex, skeletal muscle mass index (SMI), body mass index (BMI), and serum creatinine (Cr) levels [8,9]. However, to date, reports investigating the clinical factors affecting PMI in patients undergoing PD are limited. Therefore, this study aimed to evaluate the PMI levels and clarify the factors associated with PMI in patients undergoing PD.

## Materials and methods

Patients’ baseline characteristics and clinical laboratory measurements

Patients who met the following criteria were enrolled: (i) age > 20 years; (ii) ESRD managed with PD; (iii) started PD at least three months before the study; (ⅳ) without receiving renal transplantation before HD, and (ⅴ) underwent abdominal CT. This retrospective study was approved by the Institutional Review Board of the Saitama Medical Center, Jichi Medical University Ethics Committee (RINS19-HEN006), Japan, and conformed to the provisions of the Declaration of Helsinki. Informed consent was not applicable because of the retrospective study. Information regarding this study was displayed on notice boards in our hospital to inform all the patients of their right to opt out.

The baseline characteristics and clinical data of the patients were collected from their medical charts, whereas data on the primary disease leading to the need for dialysis were extracted from their medical records. Blood pressure (BP) was regularly measured in the seated position during outpatient visits, and body weight (BW) was measured in the standing position or seated position using a wheelchair. BMI was calculated using BW and body height. Blood samples were collected at ambient temperature from native veins in the outpatient department, and a CT scan was performed. The peritoneal equilibration test category was evaluated within three months before and after performing CT. The walkable state was defined as the state of walking without assistance, that is, walking without a cane or wheelchair.

PMI measurement

**Figure 1:**
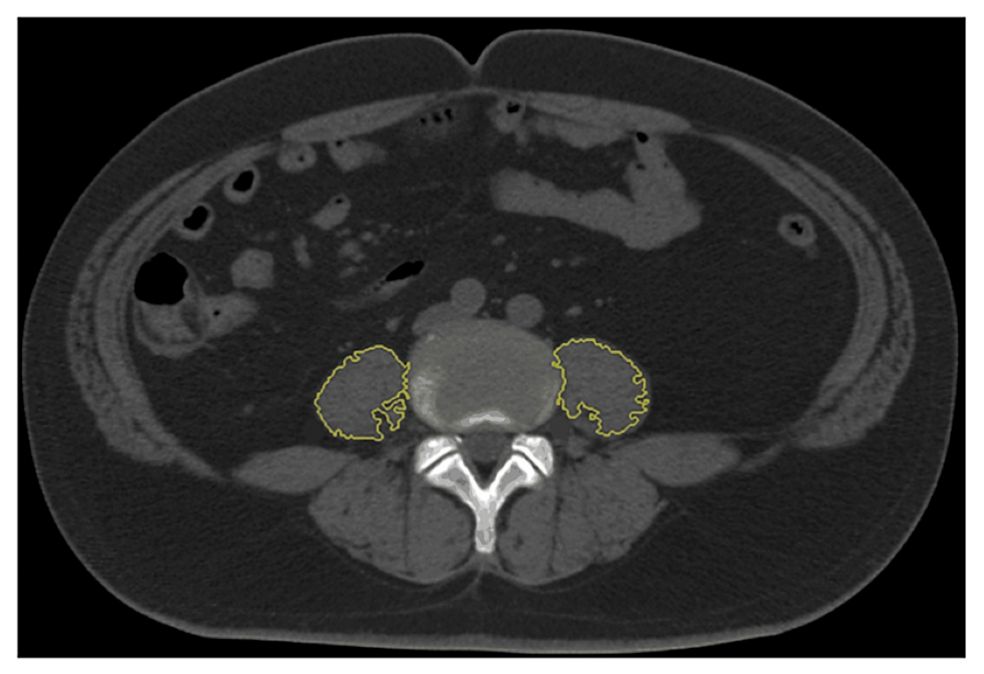
Measurement of psoas muscle area on CT Measurement of psoas muscle area at the third lumbar spine level on CT. The psoas muscle borders were manually outlined (manual trace method). Each PMI was calculated using right and left areas enclosed in line.

An annual evaluation using a multi-detector row CT scanner was performed to screen for acquired cystic renal disease and renal carcinoma in patients undergoing PD. Using these imaging techniques, psoas muscle evaluation was also performed at the third lumbar (L3) spine level to measure the cross-sectional areas of the bilateral psoas muscles (Figure 1), as previously reported [6-10]. In this study, the psoas muscle areas were measured using the manual trace method [8], and it was performed by a specific radiologist using the RapideyeCore (Canon Medical Systems Inc, Tochigi, Japan). This method was previously reported to have high intra-rater reliability and inter-rater reliability [8], and therefore, PMI evaluation in this study would guarantee replicability and reproducibility. Based on the results, PMI was calculated by normalizing the cross-sectional areas for height (cm2/m2).

Statistical analysis

Data are expressed as mean ± standard deviation or median and interquartile range. In the data analysis, we first determined whether the data were normally distributed using the Shapiro-Wilk test. The correlation between the PMI and clinical parameters was evaluated using Pearson’s correlation or Spearman’s rank correlation for normally and non-normally distributed data, respectively. Multivariable linear regression analysis was performed to identify the independent factors for PMI in patients undergoing PD. All analyses were performed using SPSS Statistics for Windows version 25.0 (IBM Corp., Armonk, NY, USA). Statistical significance was set at p < 0.05.

## Results

**Figure 2:**
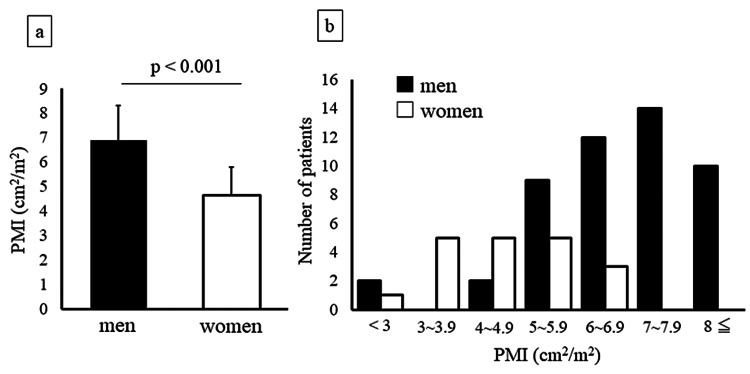
Mean PMI values by gender and number of patients undergoing PD a) Mean PMI values by gender in patients undergoing PD b) Number of patients each PMI by gender Abbreviations: PD, peritoneal dialysis; PMI, psoas muscle mass index

The general characteristics of the patients are summarized in Table 1. Sixty-eight patients undergoing PD (49 males (72%); age, 66 (54-74) years; PD duration, 25 (13-45) months) were recruited. The mean PMI of all included patients was 6.3 ± 2.0 cm^2^/m^2^. The mean PMI of men was significantly higher than that of women (6.9 ± 1.8 cm^2^/m^2^ vs. 4.6 ± 1.1 cm^2^/m^2^, p < 0.001), as shown in Figure 2A. In addition, the PMI value of men was distributed on large side compared with that of women (Figure 2B).

**Table 1:** Patients’ characteristics Abbreviations: APD: automated peritoneal dialysis; BMI: body mass index; BP: blood pressure; BUN: blood urea nitrogen; Ca: calcium; CAPD: continuous ambulatory peritoneal dialysis; CCPD: continuous cycling peritoneal dialysis; Cr: creatinine; CRP: C-reactive protein; D/P Cr: ratio of 4 hours’ dialysate to plasma creatinine; D/D0 glucose: ratio of dialysate glucose at 4 hours' dwell time to dialysis glucose at 0 dwell time; ESA: erythropoiesis-stimulating agent; P: phosphate; PD: peritoneal dialysis; PET: peritoneal equilibration test; PMI: psoas muscle mass index; PTH: parathyroid hormone. Categorical data are presented as numbers (%), while continuous data are presented as mean ± standard deviation or median (interquartile range). #Spearman’s rank correlation for skewed distributed data.

	Mean (median)
PMI, cm^2^/m^2^	6.3 ± 2.0
Gender male, n (%)	49 (72)
Age, years	66 (54-74)
PD duration, months	25 (13-45)
Reason for dialysis, n (%)	
Diabetes mellitus	24 (35)
Nephrosclerosis	17 (25)
Chronic glomerulonephritis	16 (24)
Others	11 (16)
PD method, n (%)	
CAPD	26 (38)
APD	10 (15)
CCPD	32 (47)
PET category, n (%)	
High	11 (16)
High Average	31 (46)
Low Average	22 (32)
Low	4 (6)
D/P Cr	0.68 ± 0.12
D/D0 glucose	0.38 ± 0.09
Urine volume, mL/day	497 ± 520
Ultrafiltration volume, mL/day	750 (402-1096)
Weekly Kt/V	1.55 ± 0.39
Medication, n (%)	
Vitamin D	38 (56)
Phosphate binder	54 (79)
Cinacalcet	8 (12)
ESA	63 (93)
Vital signs and physical findings	
Systolic BP, mmHg	138 ± 27
Diastolic BP, mmHg	75 ± 17
BMI, kg/m^2^	23.3 (20.5-25.7)
Walkable state, n (%)	56 (82)
Laboratory findings	
Albumin, g/dL	3.0 ± 0.7
CRP, mg/dL	0.21 (0.06-0.89)
corrected Ca, mg/dL	9.3 ± 0.6
P, mg/dL	5.4 ± 1.4
BUN, mg/dL	56 ± 16
Cr, mg/dL	11.1 ± 3.4
Hemoglobin, g/dL	10.6 ± 1.4
β_2_-microglobrin, mg/L	29.6 ± 8.4
intact PTH, pg/mL	209 (108-339)

Simple linear regression analyses were performed to investigate the correlation between PMI and clinical parameters (Table 2). PMI significantly and positively correlated with gender (r = 0.259, p < 0.001), the use of vitamin D (r = 0.365, p = 0.002), the use of phosphate binder (r = 0.283, p = 0.019), systolic BP (r = 0.361, p = 0.002), diastolic BP (r = 0.405, p = 0.001), BMI (r = 0.400, p = 0.001), walkable state (r = 0.335, p = 0.005), serum albumin (Alb) concentration (r = 0.481, p < 0.001), serum phosphate concentration (r = 0.319, p = 0.008), blood urea nitrogen concentration (r = 0.436, p < 0.001), and serum Cr concentration (r = 0.527, p < 0.001), while it significantly and negatively correlated with age (r = -0.252, p = 0.038). Furthermore, a multivariable linear regression analysis was performed using significant factors in the simple linear regression analyses. Male sex (standardized coefficient: 0.331), BMI (standardized coefficient: 0.283), serum Cr concentration (standardized coefficient: 0.289), serum Alb concentration (standardized coefficient: 0.235) and the use of vitamin D (standardized coefficient: 0.19) were independently and significantly associated with PMI (Table 2).

**Table 2:** Correlation between PMI and clinical parameters and multivariable linear regression analysis for PMI in patients undergoing PD Abbreviations: BMI: body mass index, BP: blood pressure; BUN: blood urea nitrogen; Ca: calcium; Cr: creatinine; CRP: C-reactive protein; D/P Cr: ratio of 4 hours’ dialysate to plasma creatinine; D/D0 glucose: ratio of dialysate glucose at 4 hours' dwell time to dialysis glucose at 0 dwell time; ESA: erythropoiesis-stimulating agent; P: phosphate; PD: peritoneal dialysis; PET: peritoneal equilibration test; PMI: psoas muscle mass index; PTH: parathyroid hormone.

Variable	Correlation		Multivariable linear regression	
	r	p	Standardized coefficient	p
Gender male, n (%)	0.521	< 0.001	0.331	< 0.001
Age, years	-0.252^#^	0.038	-	-
PD duration, months	-0.088^#^	0.477	-	-
Diabetes mellitus	0.194^#^	0.113	-	-
D/P Cr	0.123	0.317	-	-
D/D0 glucose	-0.127	0.304	-	-
Urine volume, mL/day	0.155	0.210	-	-
Ultrafiltration volume, mL/day	-0.005^#^	0.969	-	-
Weekly Kt/V	0.117	0.398	-	-
Vitamin D	0.365	0.002	0.195	0.017
Phosphate binder	0.283	0.019	-	-
Cinacalcet	0.193	0.114	-	-
ESA	-0.093	0.449	-	-
Systolic BP, mmHg	0.361	0.002	-	-
Diastolic BP, mmHg	0.405	0.001	-	-
BMI, kg/m^2^	0.400^#^	0.001	0.283	0.001
Walkable state, n (%)	0.335	0.005	-	-
Albumin, g/dL	0.481	< 0.001	0.235	0.007
CRP, mg/dL	-0.053^#^	0.669	-	-
corrected Ca, mg/dL	0.043	0.728	-	-
P, mg/dL	0.319	0.008	-	-
BUN, mg/dL	0.436	< 0.001	-	-
Cr, mg/dL	0.527	< 0.001	0.289	0.001
Hemoglobin, g/dL	0.074	0.550	-	-
β_2_-microglobrin, mg/L	-0.186	0.128	-	-
intact PTH, pg/mL	-0.148^#^	0.229	-	-

## Discussion

PMI has recently been evaluated in various clinical settings, such as cardiac surgery or liver and kidney transplantation [6,11-13]. However, there are limited reports on PMI in patients undergoing dialysis [7-10], and to our knowledge, there are no studies regarding PMI in patients undergoing PD. In this study, PMI was independently and significantly associated with sex, BMI, serum Cr concentration, serum Alb concentration, and the use of vitamin D.

In the Japanese population, PMI was reported to be lower in patients undergoing HD (4.8-5.1 cm^2^/m^2^) than in non-dialyzed patients (7.1-7.4 cm^2^/m^2^) [7,8,12-14]. Referring to the results of this study, PMI values in patients undergoing PD may range between those in patients undergoing HD and those in non-dialyzed patients. Although the psoas muscle was measured at a one-slice cross-sectional area at the L3 level, it would be considered to reflect the skeletal muscle mass indicating whole-body muscle, as PMI positively correlated with SMI [8,9,12]. Thus, PMI measurements using CT can offer new useful information on muscle mass, and medical staff can detect sarcopenia in medical facilities without performing dual-energy X-ray absorptiometry or bioelectrical impedance analysis.

BMI or serum Alb concentration could reflect the nutritional status or prognosis in patients with ESRD, and higher BMI was reportedly associated with better prognosis in patients with HD, called as reverse epidemiology [15]. Yajima et al. previously reported that BMI was positively associated with muscular mass, in addition to fat tissue [16]. Thus, a higher BMI would reflect physical health and good nutrition in patients undergoing HD. Furthermore, PMI positively correlated with BMI [8] and HD patients with higher PMI had better prognosis, similar to the BMI [17]. Regarding serum Alb concentration in patients undergoing dialysis, serum Alb variability was larger when maintenance HD patients were close to death [18]. Throughout the aging or death process, when nutritional status worsened or bad illness occurred, dialysis patients could be exposed to malnutrition or chronic inflammation, which is protein-energy wasting (PEW) [19]. Furthermore, chronic inflammation or increasing cytokine would cause both decreased Alb synthesis in the liver and hyper-catabolism [19]. As a result, serum Alb levels deteriorate, and sarcopenia develops. Actually, HD patients suffering from malnutrition had reportedly low BMI and SMI [20]. Therefore, maintenance of BMI and serum Alb concentration would show an important association with the prevention of sarcopenia. Serum Cr was also identified as an independent factor of PMI in patients undergoing PD, similar to those undergoing HD [8]. In general, uremic toxins including serum Cr could induce PEW [19]; therefore, it is important to manage uremia by adequate dialysis therapy. However, since Cr is a muscle-derived metabolite and its excretion would decrease in patients with ESRD, serum Cr would be proportional to muscle mass. Among patients with CKD, patients with increased skeletal muscle had high serum Cr levels, which may reportedly reflect muscle mass [21,22]. Based on the results of this study, serum Cr levels may reflect muscle mass represented by PMI in patients undergoing PD. Therefore, serum Cr would be an index for indicating muscle mass, as well as an index for indicating uremic toxins and renal function.

Furthermore, the use of vitamin D was also found to be significantly associated with PMI in this study. In patients with CKD, the frequency of vitamin D deficiency would increase with CKD progression [23], which could be caused by various factors, such as increased fibroblast growth factor 23 level, reduced vitamin D receptor, or the accumulation of uremic toxin [24,25]. In the general population, patients with sarcopenia reportedly have lower blood 25-hydroxyvitamin D (25 (OH) D) concentration [26]. In patients with CKD, vitamin D deficiency via hyperparathyroidism or changes in myokine may induce sarcopenia by decreasing muscle mass [23]. Actually, 1,25-dihydroxyvitamin D levels have been associated with skeletal muscle mass in patients with moderate CKD [27], and 25 (OH) D levels would also have been associated with muscle mass in patients undergoing PD [28]. Additionally, treatment with active vitamin D was associated with greater muscle size and strength in this cohort of patients undergoing HD [29]. In this study, the use of vitamin D might increase vitamin D levels and help to control secondary hyperparathyroidism, which might have aided in maintaining the muscle mass. However, vitamin D levels were not measured as this study is retrospective, and therefore, the direct association between vitamin D levels and muscle mass could not be commented on.

The present study has several limitations. First, the sample size is relatively small. Second, measurement software that could automatically analyze the psoas muscle was not used. However, the measurement error regarding the manual trace method was minimized and evaluated by a specific radiologist. Third, vitamin D levels were not measured, because this study was retrospectively performed. Fourth, because this study was retrospective, we could not evaluate PMI in non-dialysis patients. Finally, because the present study was cross-sectional, the directionality of the association between PMI and significantly associated clinical factors could not be clarified and the relationship between PMI and patients’ prognosis was not evaluated. Therefore, further studies are needed to clarify the clinical implications and significance of PMI in patients undergoing PD in the future.

## Conclusions

PMI was independently and significantly associated with gender, BMI, and serum Cr concentration, serum Alb concentration and the use of vitamin D. Further prospective studies are needed to clarify whether the maintenance of nutritional status or vitamin D administration could affect muscle mass in patients undergoing PD.
